# Speech understanding in diffuse steady noise in typically hearing and hard of hearing listeners

**DOI:** 10.1371/journal.pone.0274435

**Published:** 2022-09-14

**Authors:** Julie Bestel, Elsa Legris, Frédéric Rembaud, Thierry Mom, John J. Galvin

**Affiliations:** 1 Audilab, Versailles, France; 2 Audilab, Tours, France; 3 Audilab, Périgueux, France; 4 Centre Hospitalier Universitaire de Clermont-Ferrand, Clermont-Ferrand, France; 5 University Hospital Center of Tours, Tours, France; 6 House Institute Foundation, Los Angeles, CA, United States of America; Hannover Medical School: Medizinische Hochschule Hannover, GERMANY

## Abstract

Spatial cues can facilitate segregation of target speech from maskers. However, in clinical practice, masked speech understanding is most often evaluated using co-located speech and maskers (i.e., without spatial cues). Many hearing aid centers in France are equipped with five-loudspeaker arrays, allowing masked speech understanding to be measured with spatial cues. It is unclear how hearing status may affect utilization of spatial cues to segregate speech and noise. In this study, speech reception thresholds (SRTs) for target speech in “diffuse noise” (target speech from 1 speaker, noise from the remaining 4 speakers) in 297 adult listeners across 9 Audilab hearing centers. Participants were categorized according to pure-tone-average (PTA) thresholds: typically-hearing (TH; ≤ 20 dB HL), mild hearing loss (Mild; >20 ≤ 40 dB HL), moderate hearing loss 1 (Mod-1; >40 ≤ 55 dB HL), and moderate hearing loss 2 (Mod-2; >55 ≤ 65 dB HL). All participants were tested without aided hearing. SRTs in diffuse noise were significantly correlated with PTA thresholds, age at testing, as well as word and phoneme recognition scores in quiet. Stepwise linear regression analysis showed that SRTs in diffuse noise were significantly predicted by a combination of PTA threshold and word recognition scores in quiet. SRTs were also measured in co-located and diffuse noise in 65 additional participants. SRTs were significantly lower in diffuse noise than in co-located noise only for the TH and Mild groups; masking release with diffuse noise (relative to co-located noise) was significant only for the TH group. The results are consistent with previous studies that found that hard of hearing listeners have greater difficulty using spatial cues to segregate competing speech. The data suggest that speech understanding in diffuse noise provides additional insight into difficulties that hard of hearing individuals experience in complex listening environments.

## Introduction

Everyday listening conditions are complex and noisy. Human listeners are able to segregate target speech from competing sounds using a variety of acoustic cues, such as the similarity between the target speech and competing sounds and the availability of spatial cues. Competing sounds may interfere with target speech due to energetic, envelope, and/or informational masking [1–5]. Energetic masking depends on the degree of spectro-temporal overlap with the target and is thought to be largely peripheral. Envelope masking depends on the degree of envelope similarity to the target, even when there is no spectral-temporal overlap, and is thought to be more central in origin. Informational masking depends on the degree of lexical interference, the similarity between the target and competing speech (e.g., talker characteristics such as sex), and is thought to be more central in origin.

Understanding of target speech is often measured with co-located maskers, a configuration which provides no spatial cues. However, spatial cues (e.g., head shadow, inter-aural time differences, inter-aural level differences, etc.) have been shown to improve segregation of target speech and maskers [6–13]. Spatial release from masking has been shown to be poorer in hard of hearing than in typically-hearing (TH) listeners [14–20]. Spatial release from masking has also been shown to poorer in older than in younger adults [16, 18]. Utilization of spatial cues to segregate target speech and maskers has been measured using various masker stimuli and sound source setups. In sound field, some studies have used 2–3 loudspeakers with target speech typically presented directly in front of the listener (0° azimuth) and maskers presented from 0° azimuth or from some other location (e.g., ±45°, ±90° azimuth, relative to the target location). Others have used more complex, multi-speaker setups such as the R-space^TM^ 8-loudpeaker system to evaluate masked speech understanding in TH and/or hard of hearing listeners [21–27]. The masking sound used in the R-space^TM^ system replicates a “cocktail party” setting, with multi-talker babble processed to come from the multi-speaker sound sources; the target and masker speech can be assigned to any of the loudspeakers. The R-space system has also been used to evaluate the effect of microphone directionality and signal processing on signal-to-noise ratios (SNRs) for hearing aid and cochlear implant systems [22].

Compared to studies that used competing speech, multi-talker babble, or cafeteria/restaurant noise, relatively few studies have examined the benefit of spatial cues using noise maskers. Spatial release from masking has been shown to be larger with competing speech than with noise maskers [10, 28, 29]. Avivi-Reich et al. [30] found better speech understanding in young TH listeners when target speech was presented from a single loudspeaker and diffuse noise was presented from three different loudspeakers. Advantages for speech understanding in diffuse noise (3-loudspeaker array) have been observed in cochlear implant listeners using beam-forming microphones and signal-processing [31, 32]. Similarly, directional microphones have been evaluated using diffuse noise in hearing aid users [22, 33].

In general, the availability of spatial cues appears to benefit segregation of target and masker speech. Compared to speech or babble maskers (which produce some degree of energetic, envelope, and/or informational masking), there is relatively little information regarding the effect of spatialized noise (i.e., energetic masking) on TH and hard of hearing listeners’ speech understanding. A 5-loudspeaker array has been recommended by the French Society of Otorhinolaryngology-Head and Neck Surgery to assess speech perception in noise with spatial cues [34]. While this loudspeaker setup is available in many hearing centers in France, speech perception in spatialized noise is not often tested. Given hard of hearing listeners’ difficulties in utilizing spatial cues for competing speech or babble (informational and/or energetic masking) [14–20], it is unclear whether such difficulties might persist in spatialized noise. Also, it is unclear how speech understanding may differ in diffuse or co-located noise and how hearing loss may interact with these noise conditions.

In the present study, unaided sentence recognition in diffuse noise were measured in 297 TH and hard of hearing listeners at 9 Audilab hearing centers using a 5-loudspeaker array. Speech understanding in diffuse noise was evaluated in light of participants’ hearing status, age at testing, sex, word recognition scores in quiet, and phoneme recognition scores in quiet. Speech understanding in diffuse or co-located noise was also compared in another 65 TH and hard of hearing listeners. The main research questions were: 1) What factors (e.g., audiometric thresholds, age at testing, word recognition scores in quiet, etc.) predict speech understanding in diffuse noise? and 2) How does speech understanding differ in diffuse versus co-located noise?

## Methods

### Participants

Participants were recruited from 9 Audilab hearing aid centers (a commercial enterprise) in the following cities: A) Audilab Versailles, B) Audilab Chartres, C) Audilab Niort, D) Audilab Tours, E) Audilab St Pryvé St Mesmin, F) Audilab Périgueux, G) Audilab La Chaussée St Victor, H) Audilab Pau, and I) Audilab Montlouis Sur Loire. The recruited hard of hearing participants were existing Audilab patients. The recruited TH participants were individuals who accompanied Audilab patients for clinical visits, as well as Audilab clinical trainees, assistants, and audiologists. Data were collected between June 2019 and December 2020. While this study period occurred during the Covid pandemic, enrollment remained high (297 participants in total). Data from hard of hearing participants were collected during routine clinical visits that were already scheduled (i.e., no additional visits were scheduled as part of the study). This was an observational study that was approved by the Comité de Protection des Personnes Nord Ouest IV (approval number: 2018 A02729 46). Participants were provided with a study information document and were informed that they could refuse to participate in or withdraw from the study if they so desired. This was a Research Implying Human Person Type 3 study (non-interventional research in which all procedures and products are within clinical standard of care, without additional or unusual procedures of diagnosis, treatment, or supervision). As such, written informed consent was not required or collected.

Inclusion criteria were adult (> 18 years old at testing), native speakers of French, with pure-tone average (PTA) across both ears < 65 dB HL, and normal otoscopy. For hard of hearing participants, only sensorineural hearing loss was allowed.

Exclusion criteria were conductive hearing loss, hearing loss due to ototoxicity (where known), pure-tone average (PTA) threshold difference across ears > 20 dB HL, and inability to understand the study and description and/or test procedures because of cognitive or language issues. For new patients (less than 6 months from diagnosis), conductive hearing loss was measured using bone conduction thresholds. For patients with more than 6 months since diagnosis, conductive hearing loss was determined according to medical history. Hearing loss due to otoxicity was excluded because we wanted to reduce sources of heterogeneity in sensorineural hearing loss, and because chemical agents may involve specific mechanisms that we did not want to include in our study sample. Across-ear asymmetry in PTA thresholds >20 dB HL was excluded because we wanted to reduce “better ear” effects when testing with diffuse noise.

### Patient demographics and audiological measures

Pure-tone air conduction thresholds were measured for each ear in each participant using headphones for audiometric frequencies 0.5, 1, 2, 4, 6, and 8 kHz. If air conduction thresholds were available less than 6 months before the clinic appointment, these were used for the study. If air conduction thresholds were collected more than 6 months before the clinic appointment, thresholds were re-collected.

Similar to recommendations by the International Bureau for Audiology (https://www.biap.org/en/), participants were divided into 4 hearing status groups according to their binaural pure-tone average (PTA) thresholds across 0.5, 1, 2, and 4 kHz: 1) TH (PTA ≤ 20 dB HL), 2) Mild hearing loss (PTA > 20 to 40 dB HL), 3) Moderate hearing loss, 1^st^ degree (Mod-1; PTA > 40 to 55 dB HL), and 4) Moderate hearing loss, 2^nd^ degree (Mod-2; PTA > 55 to 65 dB HL). Note that the cutoff for the Mod-2 group was lower than recommended by the International Bureau for Audiology (70 dB HL) because we did not want the maximum speech level during testing to be overly high. During testing, the noise was fixed at 65 dBA and the speech level was adjusted to a maximum of 85 dBA (see details below). The present hearing status classifications are also similar to those reported by the Global Burden of Disease Expert Group on Hearing Loss [35].

A total of 335 participants were enrolled in the study. However, data from 21 participants were excluded due to loudspeaker failure from one center (Audilab Chartes), and data were excluded from another 17 participants for whom data collection was incomplete, or for whom SRTs ≤ 20 dB SNR could not be obtained within test runs (see description of test methods below). This left 297 participants that were included in the data analyses. Table 1 shows the distribution of participants for each subject group and test site, in terms of sex, age at testing, binaural PTA threshold, word recognition scores in quiet, and phoneme recognition scores in quiet.

**Table 1:** Demographic information within and across the different hearing status groups and test sites. Data are shown for the number of male and female participants, the total number of participants, mean (and standard deviation) age at testing, pure-tone average (PTA) threshold in dB HL, percent correct word recognition scores (WRS) in quiet, and percent correct phoneme recognition scores (PRS) in quiet.

Group	Site	M	F	Total	Age test	PTA	WRS	PRS
**TH**	Versailles	7	4	11	55.1±12.4	16.0±4.0	99.7±0.8	99.9±0.3
	Chartres	4	0	4	54.2±22.7	13.0±6.6	99.3±1.5	99.8±0.5
	Niort	1	0	1	40.7±0.0	16.3±0.0	100±0.0	100±0.0
	Tours	2	0	2	40.7±5.5	7.3±0.4	98.5±2.1	99.5±0.7
	St Pryvé St Mesmin	9	10	19	38.1±17.9	9.3±5.8	98.8±1.5	99.6±0.5
	Périgueux							
	La Chaussée St Victor	8	3	11	41.1±13.9	6.0±3.9	100±0.0	100±0.0
	Pau	0	1	1	54.6±0.0	11.3±0.0	100±0.0	100±0.0
	Montlouis Sur Loire	6	6	12	49.5±15.6	13.4±4.1	100±0.0	100±0.0
	Total/mean	37	24	61	44.5±17.3	11.3±5.8	99.5±1.1	99.8±0.4
**Mild**	Versailles	4	0	4	62.5±8.0	30.0±6.0	95.6±3.8	98.5±1.3
	Chartres	0	2	2	63.8±12.3	30.3±0.4	92.6±10.4	93.0±9.9
	Niort	5	7	12	62.0±15.1	32.9±6.5	92.9±7.4	97.2±3.2
	Tours	7	4	11	62.5±18.2	33.6±4.5	90.4±11.2	95.5±6.0
	St Pryvé St Mesmin	6	9	15	67.1±12.2	32.8±5.6	74.1±19.7	88.1±19.7
	Périgueux	4	2	6	65.8±14.0	35.8±3.6	90.2±6.6	96.7±2.1
	La Chaussée St Victor	5	4	9	63.9±10.6	29.4±3.4	96.4±3.5	99.1±1.1
	Pau	12	7	19	68.2±10.4	33.9±4.6	94.0±6.4	97.4±3.3
	Montlouis Sur Loire	4	5	9	68.4±10.6	29.1±6.1	100±0.0	100±0.0
	Total/mean	47	40	87	65.5±12.5	32.4±5.3	90.6±12.8	95.8±6.3
**Mod-1**	Versailles	7	4	11	74.0±9.4	46.9±4.7	81.0±14.4	90.9±9.1
	Chartres	7	4	11	74.2±5.7	48.4±4.5	51.6±23.5	72.5±23.5
	Niort	9	10	19	72.2±6.9	46.6±4.8	69.5±19.3	87.7±9.1
	Tours	15	14	29	73.3±12.2	48.3±4.7	69.0±17.9	82.9±10.6
	St Pryvé St Mesmin	7	6	13	74.6±13.8	49.1±5.0	30.6±17.4	52.9±20.3
	Périgueux	8	8	16	74.3±12.0	48.6±4.6	75.0±13.2	88.7±7.5
	La Chaussée St Victor	4	1	5	72.0±20.0	49.8±4.4	71.2±14.8	87.6±5.4
	Pau	1	0	1	70.9±0.0	40.6±0.0	88.2±0.0	96.0±0.0
	Montlouis Sur Loire	6	2	8	70.2±14.0	47.3±4.4	69.0±24.5	82.8±16.3
	Total/mean	64	49	113	73.5±11.1	47.9±4.7	66.2±21.7	81.9±14.4
**Mod-2**	Versailles	3	0	3	85.1±4.0	62.7±3.4	35.3±26.5	60.8±28.5
	Chartres							
	Niort	1	1	2	67.6.1±8.2	57.2±1.3	67.6±0.0	87.5±0.7
	Tours	9	7	16	82.4±6.5	59.9±3.0	38.2±13.9	52.8±16.9
	St Pryvé St Mesmin	1	2	3	86.5±4.4	57.7±0.7	9.8±9.0	22.5±21.0
	Périgueux	2	6	8	76.9±8.5	58.3±2.1	50.4±13.6	69.3±12.8
	La Chaussée St Victor							
	Pau	1	1	2	73.0±4.5	62.5±0.9	38.2±20.8	59.5±21.9
	Montlouis Sur Loire	1	1	2	79.8±1.3	56.9±0.0	33.8±39.5	62.0±21.2
	Total/mean	18	18	36	80.3±7.7	59.4±2.9	39.7±19.3	57.4±21.3
**All**	Versailles	21	8	29				
	Chartres	11	6	17				
	Niort	16	18	34				
	Tours	33	25	58				
	St Pryvé St Mesmin	23	27	50				
	Périgueux	14	16	30				
	La Chaussée St Victor	17	8	25				
	Pau	14	9	23				
	Montlouis Sur Loire	17	14	31				
	Total	166	131	297				

TH = typically hearing; Mild = mild hearing loss; Mod-1 = moderate hearing loss 1; Mod-2 = moderate hearing loss 2

### Speech measures

#### Word and phoneme recognition in quiet

Unaided monosyllable word recognition in quiet was measured in sound field at 65 dBA using monosyllable words from Lafon [36, 37]. Only binaural word recognition was measured. Stimuli were presented from the audiometer connected to a single loudspeaker at 0° azimuth), which was 1 m away from the listener. Two lists of 17 words each were tested for each participant. During testing, a word from the list was presented to the participant, who was asked to repeat what they heard as accurately as possible. The experimenter scored each phoneme that was correctly identified, as well as each whole word correctly identified. Scores were averaged across the two lists. Mean word and phoneme recognition scores for each hearing status group and each study site are shown in Table 1.

#### Sentence recognition in diffuse noise

**Fig 1:**
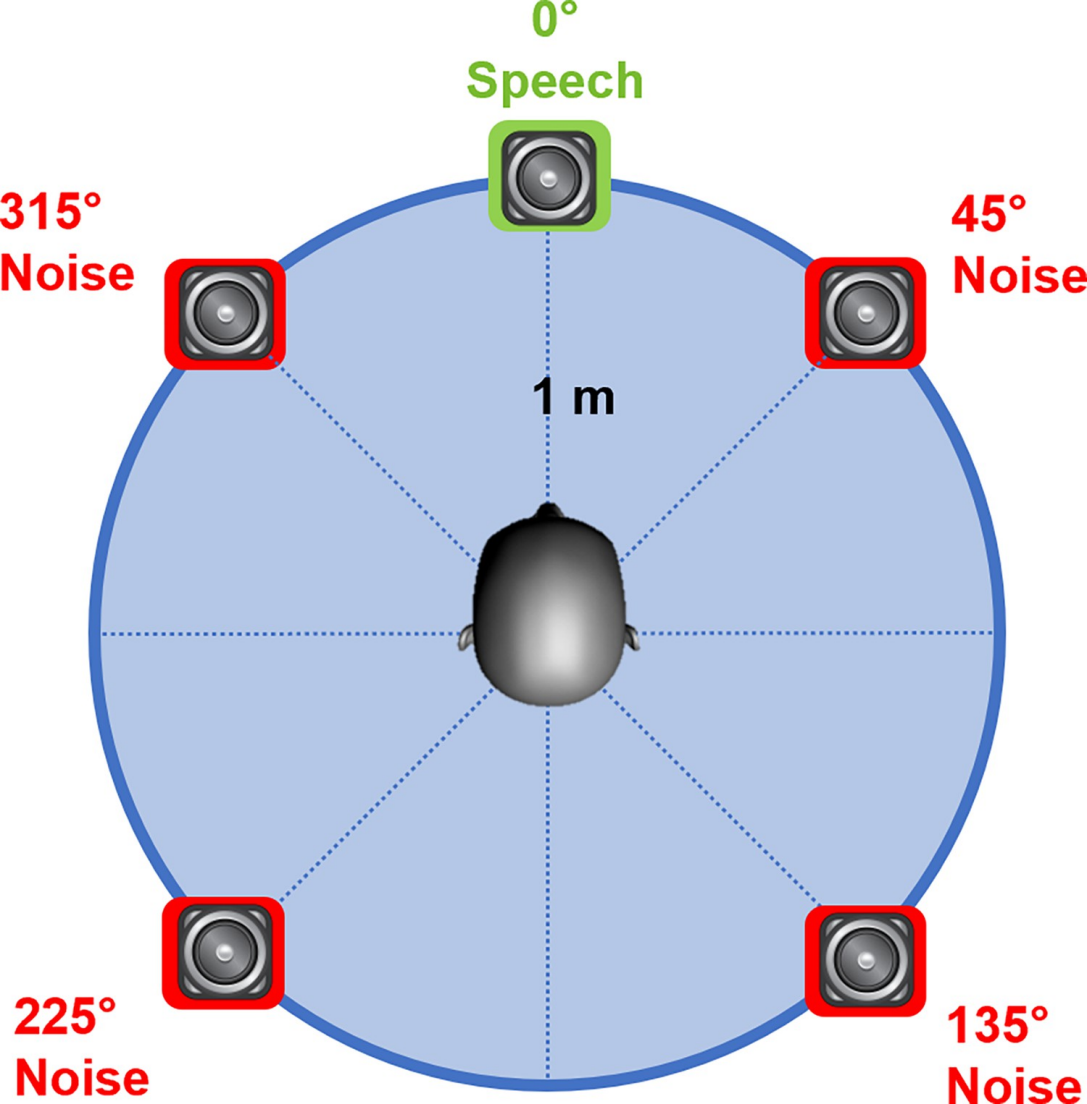
Illustration of the test setup to measure speech understanding in diffuse noise. Speech came from 0° azimuth (green speaker) and noise came from 45°, 135°, 225°, and 315° azimuth (red speakers) located 1 m away from the center of the listener’s head.

Unaided sentence recognition in noise was measured in sound field using the French Matrix stimuli from Jansen et al. [38], as implemented in the Hortech FraMatrix audiological testing software (Framatrix instruction manual, v. 1.5.4.0). Only binaural performance was measured. The French Matrix stimuli consist of 280 sentences constructed from 10 words selected from each of 5 categories (Name, Verb, Number, Noun, Adjective); the 50 words were selected to represent the distribution of phonemes in French language. The sentences are distributed into 28 lists of 20 sentences each. The sentences lists are balanced in terms of intelligibility in noise. The French Matrix test is typically implemented with speech and noise coming from a single loudspeaker directly in front of the participant (0° azimuth) who is seated 1 m away from the speaker. In the present study, speech was presented from a single loudspeaker directly in front of the participant (0° azimuth), but noise was simultaneously presented from 4 loudspeakers (45°, 135°, 225°, and 315° azimuth), creating a “diffuse noise”. Note that the same noise was presented from each of the 4 loudspeakers. The participant was seated in the middle of the speaker array, 1 m away from each speaker (see Fig 1). Stimuli were presented from an audiometer connected to the 5 loudspeakers. The target speech was delivered from one output channel of the audiometer, and the noise was delivered to the other output channel. An audio mixer was used to control the output levels of the noise fed to each of the four noise loudspeakers (see Fig 1). The steady noise was spectrally matched to the long-term average spectrum of the target sentences, and was supplied along with the FraMatrix sentences as part of the clinical test battery (“FraMatrix noise”). The overall noise level was fixed at 65 dBA. The level of the noise from each speaker was attenuated by 3 dB to maintain the fixed 65 dBA noise level; the multi-speaker 65 dBA noise level was confirmed by calibration at the listener head position. Noise was continuous during sentence recognition testing. During testing, a sentence was played in noise and presented to the participant, who was asked to repeat the sentence as accurately as possible (open-set test paradigm); the experimenter scored the correct responses. The level of the speech was adjusted according to the correctness of response. The initial SNR was +10 dB (i.e., speech presented at 75 dBA and noise at 65 dBA). If the participant repeated 3 out of 5 words correctly, the speech level was reduced; if not, the speech level was increased. The step size was automatically adjusted by the software (i.e., large steps at the beginning of the test and small steps near the end of the test). If the SNR exceeded 20 dB (i.e., speech level = 85 dBA) during a test run, the test run was discarded. The final SNR after a minimum 6 reversals in speech level was recorded as the speech reception threshold (SRT), defined as the SNR required to produce 50% correct sentence recognition in noise. Three lists of 20 sentences each were tested for each participant; test lists were randomized across participants.

#### Sentence recognition in co-located noise

SRTs were measured in co-located noise for an additional 65 participants (13 TH, 22 Mild, 19 Mod-1, and 11 Mod-2) using the same methods as described above with diffuse noise, except that speech and noise were co-located. Participants were tested while facing the center loudspeaker directly in front (0° azimuth).

### Data analysis

Data were validated by an independent contract research organization that was not part of Audilab. In the electronic case report forms, there were automatic data entry controls that prohibited experimenters from entering inappropriate values. Once data collection was complete, the dataset was “frozen” by the contract research organization, after which new data could not be entered. Data were reviewed by a data manager and a clinical research associate within the contract research organization, who checked the integrity of the data and issued queries for investigators to address.

Where appropriate, linear mixed models, Pearson correlations, linear regressions, and forward stepwise linear regression were performed using SigmaPlot (v.14) and SPSS software (v.22). Bonferroni corrections were applied to post-hoc pairwise comparisons to correct for multiple comparisons. The threshold for significance was p < 0.05.

## Results

### SRTs in diffuse noise

As per instructions provided by Hortech, and consistent with clinical practice, three lists were tested to measure SRTs. The first two runs are considered “practice” lists, and the third run is used as the SRT. Linear mixed-model analysis was performed on the SRT test run data, with group (TH, Mild, Mod-1, Mod-2) and test run (1, 2, 3) as fixed factors and participant as the random factor. Results showed significant effects for group [F(3, 293) = 141.0, p < 0.001] and test run [F(2, 551) = 250.0, p < 0.001]; there was a significant interaction [F(6, 551) = 16.5, p < 0.001]. Post-hoc Bonferroni-corrected pairwise comparisons showed that for the TH and Mild groups, SRTs were significantly higher (poorer) for run 1 than for runs 2 and 3 (p < 0.05 for both comparisons), with no significant difference between runs 2 and 3. For the Mod-1 and Mod-2 groups, SRTs were significantly higher for run 1 than for runs 2 and 3 (p < 0.05 for both comparisons), and significantly higher for run 2 than for run 3 (p < 0.05). Consistent with Hortech instructions and clinical practice, only data from the third run was used for SRTs.

**Fig 2:**
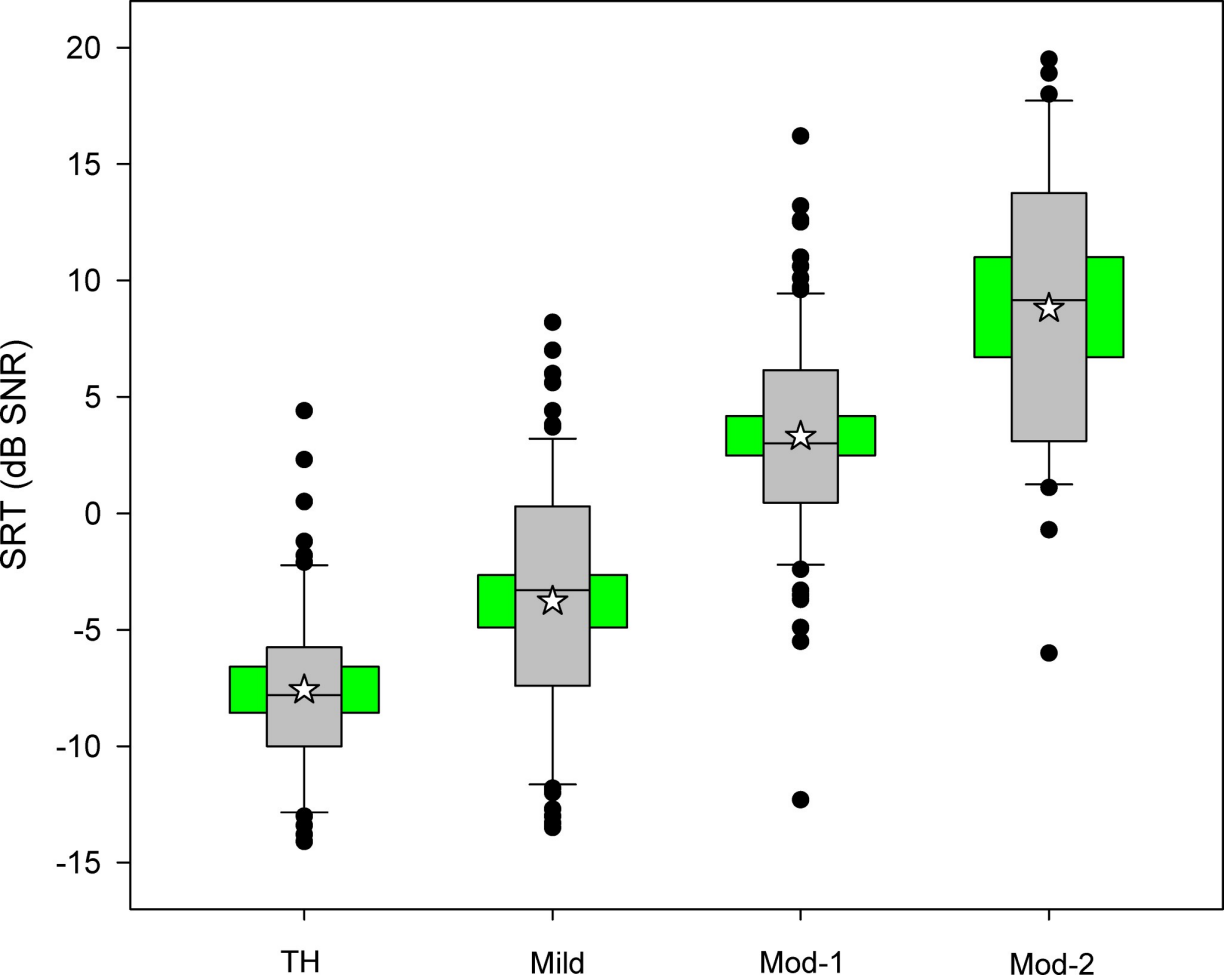
Boxplots of SRTs in diffuse noise for each hearing group. The grey boxes show the 25^th^ and 75^th^ percentile, the error bars show the 10^th^ and 90^th^ percentiles, the circles show outliers, the horizontal lines in the box show the median, the white stars show the mean, and the green boxes show the 95% confidence interval.

Fig 2 shows boxplots of SRTs in diffuse noise for the different hearing status groups. The mean SRT was -7.6 ± 3.9, -3.8 ± 5.3, 3.3 ± 4.5, and 8.8 ± 6.3 dB SNR for the TH, Mild, Mod-1, and Mod-2 groups, respectively. Linear mixed-model analysis was performed on the SRT data, with hearing group as the fixed factor and participant as the random factor. Results showed a significant effect of hearing group [F(3, 293) = 121.6, p < 0.001). Post-hoc Bonferroni pairwise comparisons showed that SRTs were significantly lower for TH group than for the Mild, Mod-1, and Mod-2 groups (p < 0.001 for all comparisons), significantly lower for Mild group than for the Mod-1 and Mod-2 groups (p < 0.001 for both comparisons), and significantly lower for the Mod-1 group than for the Mod-2 group (p < 0.001).

Table 2 shows the mean and range of PTA thresholds, age at testing, word recognition scores, phoneme recognition scores, and SRTs, as well as the number of male and female participants for the different hearing status groups. Results of various linear mixed-model analyses performed on the data are shown at right. Across all groups, there was no significant difference in the number of male and female participants. While there was substantial overlap, age at testing, word recognition scores, and SRTs differed significantly among all groups (p < 0.05 for all comparisons). Phoneme recognition scores were significantly higher for the TH and Mild groups than for the Mod-1 and Mod-2 groups (p < 0.05 for all comparisons), and significantly higher for the Mod-1 group than for the Mod-2 group (p < 0.05), with no significant difference between the TH and Mild groups.

**Table 2:** Mean and range of PTA thresholds, age at testing, word recognition scores (WRS), phoneme recognition scores (PRS), and SRTs for the different hearing status groups. Sex data are also shown. Results from linear mixed-model analyses are shown at right. For the analyses, PTA thresholds, age at testing, word recognition scores, phoneme recognition scores, and SRTs were the dependent variables, hearing status group was the fixed factor, and participant was the random factor, except for sex, where sex was the dependent variable and participant was the random factor. Significant effects are indicated by asterisks and italics. Significant post-hoc Bonferroni-adjusted pairwise comparisons are shown at far right (p < 0.05).

	**Group**	**Mean (dB HL)**	**Range (dB HL)**	**dF, res**	**F**	**p**	**Post-hoc (p < 0.05)**
PTA	TH	11.3	0.6–20.0	3, 293	1007.1	*<0*.001*	Mod-2 > Mod-1 > Mild > TH
	Mild	32.5	21.3–40.0				
	Mod-1	50.0	40.6–55.0				
	Mod-2	59.4	56.3–65.0				
	**Group**	**Male (n)**	**Female (n)**	**dF, res**	**F**	**p**	**Post-hoc (p < 0.05)**
Sex	TH	37	24	1, 295	1.2	0.280	
	Mild	47	40				
	Mod-1	64	49				
	Mod-2	18	18				
	**Group**	**Mean (years)**	**Range (years)**	**dF, res**	**F**	**p**	**Post-hoc (p < 0.05)**
Age at testing	TH	45.6	18.1–81.3	3, 293	80.4	*<0*.001*	Mod-2 > Mod-1 > Mild > TH
	Mild	65.5	19.3–88.8				
	Mod-1	73.5	37.3–93.3				
	Mod-2	80.3	61.8–90.4				
	**Group**	**Mean (% correct)**	**Range (% correct)**	**dF, res**	**F**	**p**	**Post-hoc (p < 0.05)**
WRS	TH	99.5	97.1–100.0	3, 290	135.6	*<0*.001*	TH > Mild > Mod-1 > Mod-2
	Mild	90.6	35.3–100.0				
	Mod-1	66.2	8.8–100.0				
	Mod-2	39.7	0.0–67.6				
	**Group**	**Mean (% correct)**	**Range (% correct)**	**dF, res**	**F**	**p**	**Post-hoc (p < 0.05)**
PRS	TH	99.8	99.0–100.0	3, 289	108.9	*<0*.001*	TH, Mild > Mod-1 > Mod-2
	Mild	95.8	69.0–100.0				
	Mod-1	81.9	23.0–100.0				
	Mod-2	57.4	0.0–88.0				
	**Group**	**Mean (dB SNR)**	**Range (dB SNR)**	**dF, res**	**F**	**p**	**Post-hoc (p < 0.05)**
SRT	TH	-7.6	-14.1–4.4	3, 289	121.6	*<0*.001*	Mod-2 > Mod-1 > Mild > TH
	Mild	-3.8	-13.5–8.2				
	Mod-1	3.3	-12.3–16.2				
	Mod-2	8.8	-6.0–19.5				

TH = typically hearing; Mild = mild hearing loss; Mod-1 = moderate hearing loss 1; Mod-2 = moderate hearing loss 2

Age at testing, PTA thresholds, word recognition scores, phoneme recognition scores, and SRTs in diffuse noise were compared using Pearson correlation analyses; complete results are shown in Table 3. For all groups, word recognition scores and phoneme recognition scores were highly correlated (p < 0.001 for all correlations). For the TH group, significant correlations were observed among age at testing, PTA thresholds, and SRTs (p < 0.005 for all correlations). For the Mild group, significant correlations were observed between word or phoneme recognition scores and age at testing, PTA thresholds, and SRTs (p < 0.001 for all correlations). For the Mod-1 group, significant correlations were observed between word or phoneme recognition scores and age at testing, PTA thresholds, and SRTs (p < 0.001 for all correlations); a significant correlation was also observed between PTA thresholds and SRTs (p < 0.001). For the Mod-2 group, SRTs were significantly correlated with age at testing PTA thresholds, word recognition scores, and phoneme recognition scores (p < 0.001 for all correlations). Across all participants, significant correlations were observed among age testing, PTA thresholds, word recognition scores, phoneme recognition scores, and SRTs (p < 0.001 for all correlations).

**Table 3:** Results of Pearson correlations analyses among age at testing, binaural PTA thresholds, word recognition scores (WRS), phoneme recognition scores (PRS), and SRTs. Results are shown within each hearing status group and across all participants. Significant relationships after Bonferroni correction for multiple comparisons (adjusted p = 0.005) are indicated by asterisks and italics.

	**PTA (n = 61)**		**WRS (n = 61)**		**PRS (n = 61)**		**SRT (n = 61)**	
**TH**	**r**	**p**	**r**	**p**	**r**	**p**	**r**	**p**
Age at test	0.66	*<0*.001*	-0.07	0.574	-0.07	0.574	0.36	*0*.004*
PTA			-0.09	0.486	-0.09	0.486	0.54	*<0*.001*
WRS					>0.99	*<0*.001*	-0.29	0.024
PRS							-0.29	0.024
	**PTA (n = 87)**		**WRS (n = 87)**		**PRS (n = 86)**		**SRT (n = 87)**	
**Mild**	**r**	**p**	**r**	**p**	**r**	**p**	**r**	**p**
Age at test	0.12	0.288	-0.31	*0*.004*	-0.32	*0*.003*	0.13	0.247
PTA			-0.40	*<0*.001*	-0.4	*<0*.001*	0.15	0.168
WRS					0.97	*<0*.001*	-0.45	*<0*.001*
PRS							-0.48	*<0*.001*
	**PTA (n = 113)**		**WRS (n = 110)**		**PRS (n = 110)**		**SRT (n = 113)**	
**Mod-1**	**r**	**p**	**r**	**p**	**r**	**p**	**r**	**p**
Age at test	0.23	0.013	-0.40	*<0*.001*	-0.37	*<0*.001*	0.26	0.006
PTA			-0.40	*<0*.001*	-0.44	*<0*.001*	0.48	*<0*.001*
WRS					0.91	*<0*.001*	-0.50	*<0*.001*
PRS							-0.52	*<0*.001*
	**PTA (n = 36)**		**WRS (n = 36)**		**PRS (n = 36)**		**SRT (n = 36)**	
**Mod-2**	**r**	**p**	**r**	**p**	**r**	**p**	**r**	**p**
Age at test	0.01	0.934	-0.40	0.015	-0.44	0.008	0.57	*<0*.001*
PTA			-0.23	0.186	-0.17	0.320	0.16	0.345
WRS					0.82	*<0*.001*	-0.54	*<0*.001*
PRS							-0.57	*<0*.001*
	**PTA (n = 297)**		**WRS (n = 293)**		**PRS (n = 293)**		**SRT (n = 297)**	
**All**	**r**	**p**	**r**	**p**	**r**	**p**	**r**	**p**
Age at test	0.70	*<0*.001*	-0.58	*<0*.001*	-0.52	*<0*.001*	-0.58	*<0*.001*
PTA			-0.74	*<0*.001*	-0.67	*<0*.001*	0.75	*<0*.001*
WRS					0.93	*<0*.001*	-0.74	*<0*.001*
PRS							-0.73	*<0*.001*

TH = typically hearing; Mild = mild hearing loss; Mod-1 = moderate hearing loss 1; Mod-2 = moderate hearing loss 2

**Fig 3:**
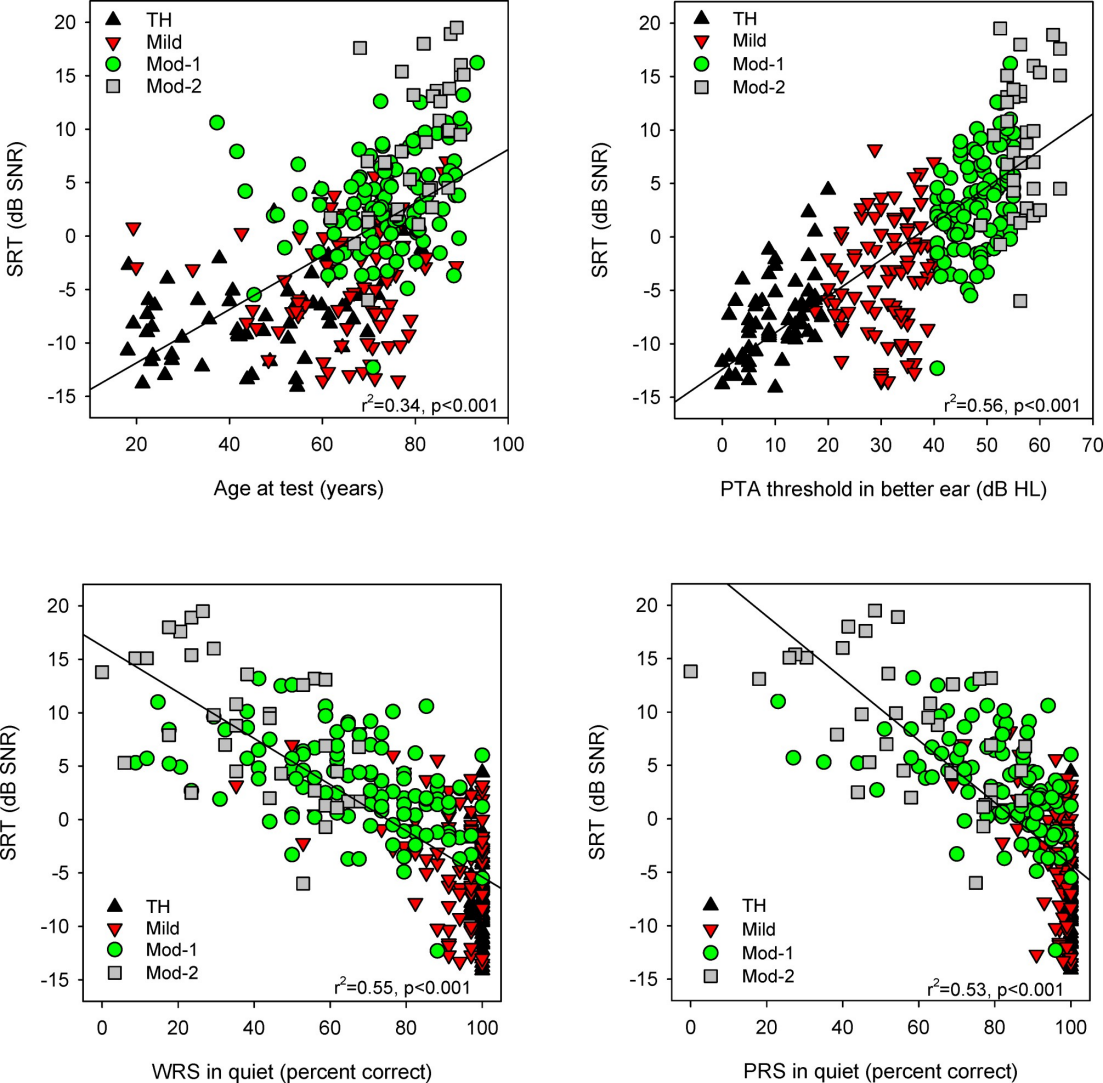
Scatterplots of SRTs in diffuse noise. SRTs are shown as a function of (clockwise from top left): age at testing, pure-tone average (PTA) threshold in the better ear, phoneme recognition scores (PRS) in quiet, and word recognition scores (WRS) in quiet. In each panel, the different symbols show data for the different hearing groups. The diagonal lines show linear regression fits to all data in the plot; r and p values are shown in the lower right of each panel.

Fig 3 shows SRTs in diffuse noise as a function of age at testing (upper left), PTA threshold in the better ear (upper right), word recognition scores (lower left), and phoneme recognition scores (lower right). Linear regression analyses across all data showed significant relationships between SRTs in diffuse noise and age at testing (r^2^ = 0.34, p < 0.001), PTA thresholds in the better ear (r^2^ = 0.57, p < 0.001), word recognition scores (r^2^ = 0.55, p < 0.001), and phoneme recognition scores (r^2^ = 0.45, p < 0.001). PTA thresholds in the better ear were significantly correlated with SRTs in diffuse noise only within the TH (r^2^ = 0.29, p < 0.001), and Mod-1 groups (r^2^ = 0.23, p < 0.001).

While the inclusion criteria required less than 20 dB inter-aural asymmetry in terms of PTA thresholds, the mean asymmetry increased across the hearing status groups: 2.3 ± 2.2, 3.6 ± 3.2, 4.6 ± 3.7, 5.7 ± 3.7 dB for the TH, Mild, Mod-1, and Mod-2 groups, respectively. A linear mixed model, with hearing status group (TH, Mild, Mod-1, Mod-2) as the fixed factor and participant as the random factor, showed significant effect of hearing status group on PTA inter-aural asymmetry [F(3, 293) = 9.9, p < 0.001). Post-hoc Bonferroni-corrected pairwise comparisons showed that asymmetry was significantly larger for the Mod-1 and Mod-2 groups than for TH or Mild groups (p < 0.001 for all comparisons), with no significant difference between the Mod-1 and Mod-2 groups, or between the TH and Mild groups. Across all participants, linear regression analysis showed that SRTs in diffuse noise were weakly (but significantly) correlated with PTA inter-aural asymmetry (r^2^ = 0.03, p = 0.002), suggesting little clinical relevance.

Forward stepwise linear regression analysis was performed on the SRT data. Predictors entered into the model included: age at testing; threshold in the better ear at 500, 1000, 2000, 4000, 6000, and 8000 Hz; PTA threshold in the better ear; word recognition scores; phoneme recognition scores. Complete results of the model are shown in Table 4. According to the criteria to enter (p < 0.05), the following four predictors were included the model: PTA thresholds in the better ear, word recognition scores, phoneme recognition scores, and threshold at 1000 Hz in the better ear. Note that while age at testing was significantly correlated with SRTs in diffuse noise, it was also significantly correlated with PTA thresholds in the better ear, word recognition scores, and phoneme recognition scores, indicating substantial co-linearity (Table 3). According to the criteria to enter, age at testing was not included in the model. The predictive value with only PTA in the better ear was large and significant (r^2^ = 0.57, p < 0.001). When PTA in the better ear was combined with word recognition scores, the predictive value increased (r^2^ = 0.65, p < 0.001). Adding phoneme recognition scores and 1000-Hz thresholds in the better ear marginally increased the predictive value (r^2^ = 0.66, p < 0.001). After consideration of co-linearity among predictors (value-inflation factor > 5), the model that included PTA threshold in the better ear and word recognition scores in quiet best explained the variability in SRTs in diffuse noise (r^2^ = 0.65, p < 0.001).

**Table 4:** Results from forward stepwise linear regression model. SRT in diffuse noise was the dependent variable. The four predictors in the model were pure-tone average (PTA) threshold in the better ear, word recognition scores (WRS) in quiet, phoneme recognition scores (PRS) in quiet, and thresholds in the better ear at 1000 Hz. Coefficients of the model are shown at left, the results from the analysis of variance (ANOVA) are shown in the middle, and the prediction (r and r^2^) are shown at right. The asterisks indicate significant effects for the coefficients and the ANOVA results. For the value-inflated factor (VIF) column, the italicized values indicate substantial collinearity among the entered predictors in the model (VIF > 5).

	Unstandardized Coefficients		Standardized Coefficients				ANOVA			Prediction	
Model	B	STE	Beta	t	p	VIF	dF, res	F	p	r	r^2^
Constant	-12.4	0.7		-18.4	*<0*.001*		1, 293	387.6	*<0*.001*	0.76	0.57
PTA	0.3	0.0	0.8	19.7	*<0*.001*						
Constant	1.6	1.8		0.9	0.365		2, 292	273.3	*<0*.001*	0.81	0.65
PTA	0.2	0.0	0.4	8.7	*<0*.001*	2.2					
WRS	-0.1	0.0	-0.4	-8.3	*<0*.001*	2.2					
Constant	3.0	1.9		1.6	0.116		3, 291	185.8	*<0*.001*	0.81	0.66
PTA	0.2	0.0	0.5	8.9	*<0*.001*	2.2					
WRS	-0.1	0.0	-0.3	-3.2	*0*.001*	*6*.*2*					
PRS	-0.1	0.0	-0.2	-2.1	*0*.035*	4.6					
Constant	3.4	1.9		1.8	0.070		4, 290	142.4	*<0*.001*	0.81	0.66
PTA	0.3	0.0	0.6	6.9	*<0*.001*	*7*.*0*					
WRS	-0.1	0.0	-0.3	-3.3	*0*.001*	*6*.*2*					
PRS	-0.1	0.0	-0.2	-2.3	*0*.021*	4.6					
T1000	-0.1	0.0	-0.2	-2.2	*0*.029*	*6*.*6*					

### SRTs in co-located vs. diffuse noise

**Fig 4:**
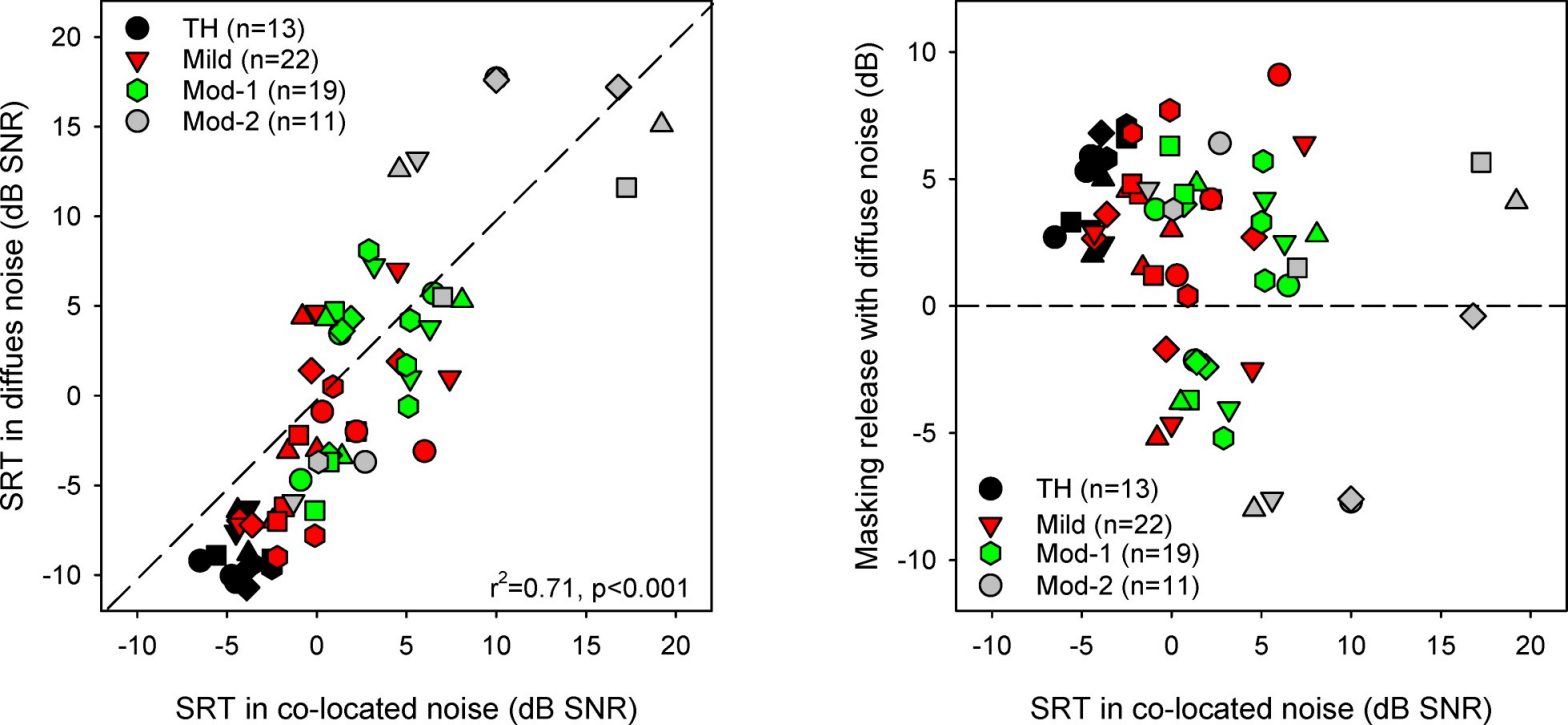
SRTs in diffuse vs. co-located noise. Left: SRTs in diffuse noise as a function of SRTs in co-located noise for the different hearing status groups. The solid diagonal line shows unity; values below the diagonal indicate that SRTs were lower with diffuse noise than with co-located noise. Right: Masking release with diffuse noise (SRTs in co-located noise—SRTs in diffuse noise) as a function of SRTs in co-located noise for the different hearing status groups. Values greater than 0 indicate masking release with diffuse noise; values less than 0 indicate interference with diffuse noise.

SRTs with co-located or diffuse noise were measured in another 65 participants. With co-located noise, the mean SRT was -4.2 ± 1.1, 0.2 ± 3.2, 2.9 ± 2.6, and 8.4 ± 7.0 dB SNR for the TH, Mild, Mod-1, and Mod-2 groups, respectively. With diffuse noise, the mean SRT was -8.9 ± 1.4, -2.4 ± 4.6, 1.9 ± 4.3, and 8.8 ± 9.2 dB SNR for the TH, Mild, Mod-1, and Mod-2 groups, respectively. The left panel of Fig 4 shows SRTs in diffuse noise as a function of SRTs in co-located noise. Linear mixed-model analysis was performed on the SRT data, with noise configuration (co-located with speech, diffuse) and hearing status group (TH, Mild, Mod-1, Mod-2) as fixed factors and participant as the random factor. Results showed significant effects for noise configuration [F(1, 61) = 15.3, p < 0.001] and hearing status group [F(3, 61) = 30.3, p < 0.001]; there was a significant interaction [F(3, 46) = 4.1, p = 0.010]. Post-hoc Bonferroni pairwise comparisons showed that SRTs were significantly lower (better) in diffuse noise than in co-located noise only for the TH (p < 0.001) and Mild groups (p = 0.003). For co-located noise, SRTs were significantly lower for the TH group than for the Mild (p = 0.043), Mod-1 (p < 0.001) and Mod-2 groups (p < 0.001), significantly lower for the Mild and Mod-1 groups than for the Mod-2 group (p < 0.001 for both comparisons), with no significant difference between the Mild and Mod-1 groups. For diffuse noise, SRTs were significantly lower for the TH group than for the Mild (p = 0.001), Mod-1 (p < 0.001) and Mod-2 groups (p < 0.001), significantly lower for the Mild than for the Mod-1 (p = 0.018) and Mod-2 groups (p < 0.001), and significantly lower for the Mod-1 than for the Mod-2 group (p < 0.001). Linear regression analysis across all data showed a significant correlation between SRTs in co-located and diffuse noise (r^2^ = 0.71, p < 0.001).

Masking release with diffuse noise was calculated as the difference between SRTs with co-located noise and SRTs with diffuse noise. The mean masking release with diffuse noise was 4.8 ± 1.8, 2.6 ± 3.7, 1.1 ± 3.8, and -0.5 ± 6.0 dB for the TH, Mild, Mod-1, and Mod-2 groups, respectively. The right panel of Fig 4 shows masking release with diffuse noise as a function of SRTs in co-located noise. As noted above, SRTs were significantly different between diffuse and co-located noise only for the TH and Mild groups, indicating that only the TH and Mild groups experienced significant masking release with diffuse noise. Linear mixed-model analysis was performed on the masking release data, with hearing status group (TH, Mild, Mod-1, Mod-2) as the fixed factor and participant as the random factor. Results showed a significant effect of group [F(3, 61) = 4.1, p = 0.010]. Post-hoc Bonferroni pairwise comparisons showed that masking release was significantly larger for the TH group than for the Mod-2 group (p = 0.011), with no significant differences among the remaining groups. Across all participants, linear regression analysis showed a weak (but significant) relationship between masking release and SRTs in co-located noise (r^2^ = 0.06, p = 0.046).

## Discussion

The main questions of this study were: 1) What factors predict speech understanding in diffuse noise? and 2) How does speech understanding differ in diffuse versus co-located noise? Regarding the first question, significant relationships were observed between SRTs in diffuse noise and PTA thresholds, age at testing, word recognition scores, and phoneme recognition scores (p < 0.001 for all correlations; Table 3; Fig 3). However, there was also great variability in terms of demographic factors, word recognition scores, phoneme recognition scores, and SRTs in diffuse noise (Table 2). For example, SRTs in diffuse noise ranged by 19.4, 21.7, 28.5, and 25.5 dB for the TH, Mild, Mod-1, and Mod-2 groups, respectively. The range for age at testing was similarly broad: 63.2, 69.5, 56.0, and 28.6 years for the TH, Mild, Mod-1, and Mod-2 groups, respectively.

### Predictors of SRTs in diffuse noise

Stepwise linear regression showed that SRTs in diffuse noise were largely predicted by a combination of PTA thresholds and word recognition scores in quiet (r^2^ = 0.66, p < 0.001). Given the predictably strong correlation between word and phoneme recognition scores (r^2^ = 0.98, p < 0.001), only one of these speech measures was needed for the model. And while age at testing was also predictive of SRTs in diffuse noise (r^2^ = 0.34, p < 0.001), it was strongly correlated with PTA thresholds (r^2^ = 0.49, p < 0.001). Thus, SRTs in diffuse noise appeared to be predicted by a combination of relatively peripheral (PTA thresholds) and central auditory processing (word recognition scores). The predictive value of PTA thresholds and word recognition in quiet is in line with Plomp’s [39] finding that speech understanding in noise in hard of hearing listeners was limited by a combination of audibility and signal distortion. Similar to the present findings, Kuhne [40] found a significant correlation between sentence recognition in noise and word recognition in quiet. However, Wilson [41] found no significant relationship between word recognition in quiet and in noise, suggesting that testing in noise may capture some different aspect of speech perception in hard of hearing listeners. Across all participants, we found a strong correlation between SRTs in diffuse noise and PTA thresholds (r^2^ = 0.56, p < 0.001) and word recognition scores (r^2^ = 0.55, p < 0.001). However, within the hearing status groups, correlations were somewhat weaker. SRTs in diffuse noise were significantly correlated with PTA thresholds in the TH (r^2^ = 0.29; p < 0.001) and Mod-1 groups (r^2^ = 0.23; p < 0.001), and with word recognition scores in the Mild (r^2^ = 0.22, p < 0.001), Mod-1(r^2^ = 0.25, p < 0.001), and Mod-2 groups (r^2^ = 0.29, p < 0.001).

### Effect of hearing loss on benefit of spatial cues

Regarding the second main research question, SRTs were significantly lower in diffuse noise than in co-located noise only for the TH and Mild groups. Similar to previous studies [14–19], the Mod-1 and Mod-2 groups benefitted less from spatial cues than did the TH and Mild groups. In adults, speech understanding and noise and spatial release from masking have been shown to worsen with increasing hearing loss [6, 7, 14, 16–18, 20], possibly due to reduced audibility of the target speech and/or suprathreshold distortion to the speech signal due to hearing loss [17, 39, 42, 43]. There was a weak but significant correlation between SRTs in co-located noise and masking release with diffuse noise (r^2^ = 0.06, p = 0.046). Taken together, the data suggest that testing with diffuse and co-located noise may reveal different deficits in speech understanding among hard of hearing listeners.

### Clinical implications

As of 2018, speech in noise testing is now mandatory in France for all auditory evaluations for hearing aid candidates and recipients [34]. While a 5-loudspeaker setup is installed in many clinical hearing centers throughout France, as recommended by the French Society of Otorhinolaryngology-Head and Neck Surgery [34], speech understanding in noise is most often measured using co-located noise. One motivation for this study was that there was great interest among Audilab centers regarding how speech understanding might differ between co-located and diffuse noise, especially in light of hearing loss. Assuming that the listener is directly facing a single loudspeaker, co-located speech and noise offers greater stimulus control, in that speech and noise will be subjected to the same room acoustics and speech and noise will be identically presented to each ear. As such, testing with co-located speech and noise involves a very simple test setup that may be preferable in the clinic. While co-located speech and noise offers good stimulus control, it does not occur in the natural world, except from electronic devices and public address systems. In everyday complex listening environments, target speech and maskers do not typically come from the same sound source. Provided that there are sufficient localization cues and abilities, a listener will typically turn to face a target talker and benefit from the spatial separation between the target and maskers [44–46]. Thus, the present target and noise speaker setup is in line with an ecologically valid listening situation after head turn. Note that the same noise was presented from the four masker speaker locations. Due to room acoustics and pinna effects, the spectrum of the noise may have deviated across speakers at the ear level [47]. Another approach that could easily be implemented would be to introduce a small delay in the noise across the speakers; this might help listeners to better localize the noise sources.

The recommended clinical French Matrix testing procedure is to measure SRTs three times, with the first two runs discarded as practice and familiarization. For all groups, SRTs for the first run were significantly poorer than for runs 2 and 3. For the TH and Mild groups, there was no significant difference between runs 2 and 3. For the Mod-1 and Mod-2 groups, SRTs were significantly poorer for run 2 than run 3. This suggests for individuals with moderate hearing loss, some learning may persist even at the third test run; it is unclear whether SRTs would stabilize with additional test runs. Note that this observation is true only for the present diffuse noise conditions where noise was presented at 65 dBA and the speech level was adapted. Further testing with a greater number of hard of hearing listeners is needed to observe whether the same holds true for co-located speech and noise.

Interestingly, SRTs in diffuse noise < 20 dB SNR were obtained in 32 participants for whom word recognition scores were < 40% correct and in 9 participants for whom phoneme recognition scores were < 40% correct, suggesting that SRTs in diffuse noise may be obtained in listeners that exhibit poor word or phoneme recognition in quiet. While the syntax is fixed, the French Matrix test offers little context; in this study, the test was administered in an open-set paradigm, further reducing context cues. In clinical practice, poor word recognition in quiet often obviates testing sentence recognition in noise. While testing with co-located speech and noise is the current clinical standard, the present data suggest that many hard of hearing listeners with poor word recognition in quiet may be capable of some degree of speech understanding in diffuse noise. If time allows, it may be worthwhile to capture speech performance for these listeners in both co-located and diffuse noise.

### Limitations to the study

There was substantial variability in age at testing within the different hearing status groups (Table 2), and age at testing was significantly correlated with SRTs in diffuse noise. Cognitive function may have limited speech performance; unfortunately, this was not measured in the present study as it is not part of the standard of clinical care. In future research and/or clinical evaluation, measures of cognitive function may help to better understand the variability in speech performance across patients. Hearing loss has been associated with cognitive decline [48, 49]. Unfortunately, hearing aid usage and compliance is typically poor in patients with cognitive impairment [50].

In this study, unaided SRTs were measured in hard of hearing listeners. Given that the maximum speech presentation level was 85 dBA (20 dB SNR) and the elevated PTA thresholds for the Mod-1 and Mod-2 groups, audibility of the target sentence may have been an issue. For the Mod-2 group, where the maximum PTA threshold was 65 dB HL and the maximum allowable SRT was 20 dB SNR (sentence presented at 85 dBA), the maximum sentence presentation level would be 20 dB sensation level (SL; difference between sentence level and PTA threshold level). This is a much lower effective sentence level than for the TH group, where the maximum PTA threshold was 20 dB HL; at a 20 dB SNR, this would correspond to a maximum sentence presentation level of 65 dB SL. It is unclear how aided hearing may affect SRTs in diffuse noise. Hearing aids would improve audibility, but device amplitude compression may effectively reduce the SNR, relative to the input SNR. Hearing aids may also distort other aspects of the signal, but hearing aid features such as noise reduction and directional microphones may improve SRTs in diffuse noise. Comparing unaided and aided SRTs in diffuse noise would be a valuable future study.

Finally, the inclusion criteria for this study required less than 20 dB inter-aural asymmetry in terms of PTA thresholds. Inter-aural asymmetry was significantly larger for the Mod-1 and Mod-2 groups than for the TH and Mild groups (p < 0.05 for all comparisons); asymmetry was also significantly correlated with SRTs in diffuse noise (p < 0.002). Because of this, PTA thresholds in the better ear were used in the stepwise linear regression model. The inter-aural asymmetry may have contributed to the somewhat large variability observed in SRTs within each of the hearing status groups (Fig 2). Reducing inter-aural asymmetry via optimal fitting of hearing aids may help to maximize hard of hearing listeners’ binaural perception of speech in spatialized noise [51–53].

## Conclusions

In this study, speech understanding in noise was measured in diffuse noise in 297 participants from 9 Audilab centers in France. Speech was delivered from the front speaker (°0 azimuth) and noise was delivered from 4 loudspeakers (45°, 135°, 225°, and 315° azimuth). In another 65 participants, SRTs were measured in diffuse noise and in co-located noise. Major findings include:

- SRTs in diffuse noise were significantly correlated with PTA thresholds, age at testing, word recognition scores in quiet, and phoneme recognition scores in quiet. A stepwise linear regression model showed that SRTs in diffuse noise were well-predicted by a combination of PTA thresholds and word recognition scores in quiet.
- SRTs in diffuse noise were significantly lower than SRTs in co-located noise only for the TH and Mild groups. For the Mild, Mod-1, and Mod-2 groups, masking release with diffuse was not significantly correlated with SRTs in co-located noise, suggesting that hearing loss limited the benefit of spatially separated speech and noise.
- Measuring speech performance in diffuse noise with unaided hearing may provide additional insight into difficulties that hard of hearing individuals experience in complex listening environments. One advantage of testing with unaided hearing is that differences in hearing aid signal processing and parameters across patients are not a factor in performance, provided there is sufficient audibility during testing. Also, diffuse noise provides spatial cues without the lexical informational masking associated with spatially separated competing speech.

## Supporting information

S1 Dataset (XLSX)
